# Assessment of Caregiver Stress Across Dementia and Non-Dementia Groups

**DOI:** 10.1093/geroni/igaf122.4071

**Published:** 2025-12-31

**Authors:** Urooj Hudda, Ethan Bradford, Gohar Azhar, Regina Gibson, Madeline Wood, Debbie Brady, Jeanne Wei

**Affiliations:** University of Arkansas for Medical Sciences, Little Rock, Arkansas, United States; University of Arkansas for Medical Sciences, Little Rock, Arkansas, United States; University of Arkansas for Medical Sciences, Little Rock, AR, LITTLE ROCK, Arkansas, United States; University of Arkansas for Medical Sciences, Little Rock, Arkansas, United States; University of Arkansas for Medical Sciences, Little Rock, Arkansas, United States; University of Arkansas for Medical Sciences, Little Rock, Arkansas, United States; University of Arkansas for Medical Sciences, Little Rock, AR, LITTLE ROCK, Arkansas, United States

## Abstract

As the U.S population ages, informal caregivers have become essential to supporting older adults. This study aims to explore the emotional, financial, and health-related stressors and challenges experienced by caregivers attending to patients living with dementia compared to those without dementia. An anonymous, cross-sectional, convenience sample survey exploring caregiver burden was administered to 114 primary caregivers. A factor analysis of 10 items related to caregiver stress with polychoric correlations was conducted and factor scores were calculated to serve as an index of caregiver stress. Missing data were handled using multiple imputation. Linear regression was conducted using factor scores to examine the relationship between caregiver burden and being a dementia caregiver as well as the number of caregivers. 37 participants were dementia caregivers and 77 were non-dementia caregivers. Most participants were over the age of 60 (59.5%). 37.2% of dementia caregivers responded that they had a stress level of “severe” or higher compared to 21.4% of non-dementia caregivers. 13.9% of dementia caregivers “often” or “always” had trouble paying their bills in the last year, compared to 5.2% of non-dementia caregivers. A linear regression concluded that dementia caregivers had significantly higher burden than those without (p = 0.040). Receiving help from other caregivers significantly reduced caregiver burden (p = 0.016). Caregivers of patients who are living with dementia experience significantly greater stress, which might be mitigated through shared responsibilities. Addressing stressors of caregivers is critical not only for their wellbeing but can also impact the health outcomes of patients and potentially delay or prevent institutionalization.

